# Transgenerational Effects of Maternal Immune Activation on Specific Antibody Responses in Layer Chickens

**DOI:** 10.3389/fvets.2022.832130

**Published:** 2022-02-17

**Authors:** Michel B. Verwoolde, Joop Arts, Christine A. Jansen, Henk K. Parmentier, Aart Lammers

**Affiliations:** ^1^Adaptation Physiology Group, Department of Animal Sciences, Wageningen University & Research, Wageningen, Netherlands; ^2^Animal Nutrition Group, Department of Animal Sciences, Wageningen University & Research, Wageningen, Netherlands; ^3^Cell Biology and Immunology Group, Department of Animal Sciences, Wageningen University & Research, Wageningen, Netherlands

**Keywords:** chicken, transgenerational, innate immunity, β-glucan, lipopolysaccharide, antibody response

## Abstract

Activation of the maternal immune system may affect innate and adaptive immune responses in the next generation and may therefore have implications for vaccine efficacy and dietary immune modulation by feed additives. However, transgenerational effects on immune responses in chickens have been investigated to a limited extend. The present study investigated effects of intratracheal (i.t) specific and aspecific immune activation of laying hens on specific antibody production in the next generation. In two experiments laying hens received intratracheally an immune stimulus with human serum albumin (HuSA) or lipopolysaccharide (LPS). In experiment 1, hatchlings of the immune activated hens were at 4 weeks i.t. immunized with HuSA or HuSA+LPS. Maternal immune activation with LPS increased HuSA specific IgY and IgM responses in offspring. These results suggest a transgenerational effect of the maternal immune system on the specific antibody response in the next generation. In experiment 2 hatchlings received either β-glucan-enriched feed or control feed and were i.t. immunized with HuSA. Maternal immune activation with LPS decreased IgY anti-HuSA responses after HuSA immunization within hatchlings that received β-glucan enriched feed. The results of Experiment 2 suggest a transgenerational link between the innate immune system of mother and specific antibody responses in offspring. Despite variabilities in the outcomes of the two experiments, the observations of both suggest a link between the maternal innate immune system and the immune system of the offspring. Furthermore, our results may imply that maternal activation of the innate immune system can influence immune modulating dietary interventions and vaccine strategies in the next generation.

## Introduction

Within chicken husbandry, the containment of infectious diseases is very important. One of the factors that might be of influence is the impact of the maternal immune system on disease resistance in the neonate, whereby activation of the maternal immune system may influence the immune system of the neonate in a transgenerational fashion. An example of these transgenerational effects is the transfer of maternal antibodies in birds that are passively transmitted to the neonate (1, 2). Another example is transgenerational inheritance, in which epigenetic mechanisms, rather than changes in the DNA code, are involved. It is hypothesized that these epigenetic mechanisms cause transgenerational effects on neonatal immunity in mice (3) and birds (4, 5). Apart from transgenerational effects on neonatal immunity, studies in mammals demonstrate an increased risk of developing stress-related problems, fertility problems, reproductive problems and higher susceptibility to metabolic disorders such as diabetes and obesity in the F_1_ and even F_2_ generations due to environmental effects in the F_0_ generation (6–8). Besides negative effects, transgenerational mechanisms may also be beneficial to the offspring. For example, immune activation with lipopolysaccharide (LPS) in pied flycatchers caused elevated antibody production in the offspring (9). These transgenerational mechanisms, may enable the mother to prepare her offspring for their future environment whereby the immune responsiveness of the offspring is affected by information of the mother in a transgenerational fashion (3, 10, 11). So far, it has not been studied whether activation of the maternal immune system in chickens influence the specific antibody response in the next generation. Therefore, the present study in laying hens aims to investigate whether maternal activation of the innate or adaptive immune system will affect the specific antibody responses in the next generation.

Laying hens were either immune activated with LPS as a microbial-associated molecular pattern (MAMP) acting as a non-specific activator of the innate immune system, or human serum albumin (HuSA) acting as a specific stimulator of the adaptive immune system. We hypothesize that the innate immune system might play an important role in transgenerational effects, which was previously proposed by Berghof et al. (5). Effects of the maternal immune activators LPS and HuSA on the antibody responses and their isotypes to HuSA in the next generation were measured in two independent experiments. Like LPS, β-glucan act via receptors present on antigen presenting cells and therefore both have the potential to influence specific antibody responses (12, 13). Specific antibody response is in the current study defined as a significant increase in the specific antibody level after an immunization with an antigen. If maternal immune activation indeed affects the immune system in the next generation, immune modulation by dietary additives might also be influenced (14–17). Experiment 2 was therefore performed to study whether maternal immune activation could also influence the effects of dietary treatment with β-glucan on antibody responses in the next generation.

## Materials and Methods

### Ethics Statement

Both experiments were approved by the Animal Welfare Committee of Wageningen University and Research in accordance with Dutch laws and regulations on the execution of animal experiments (Experimental Codes: 2013076.d and 2014057).

### Experimental Design

**Figure 1:**
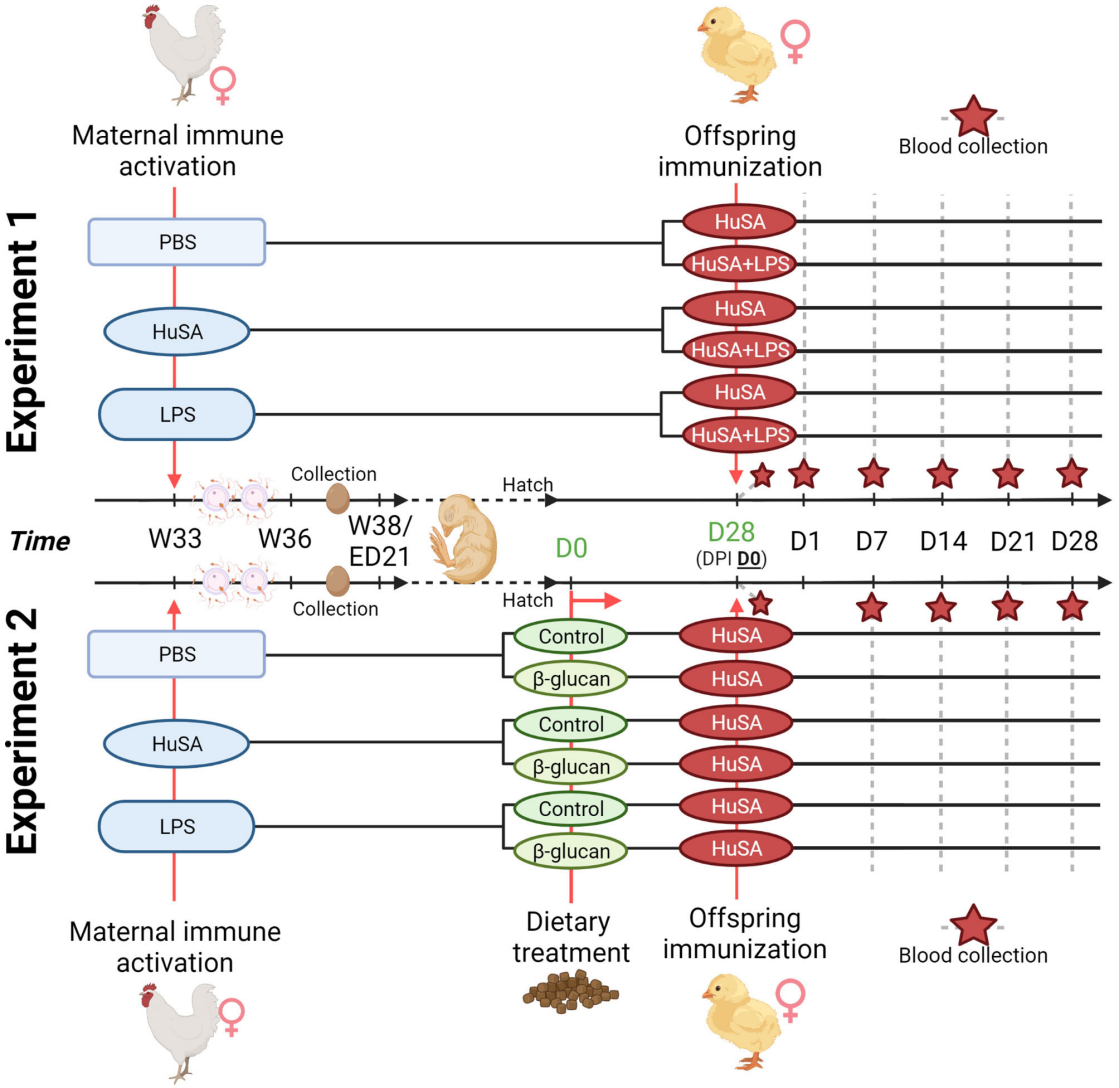
Timeline of the two transgenerational experiments. For both experiments, maternal immune activation with PBS, HuSA and LPS was performed on 33-week-old hens (W33). Hens were inseminated with pooled sperm at the day of immunization and repeated 2 weeks later. Eggs were collected when the hens reached the age of 36 weeks (W36) until 38 weeks of age (W38). For experiment 1, hatched chicks received at 28 days of age (DPI D0), the immunization with HuSA or HuSA+LPS. Blood was collected at DPI: D0, D1, D7, D14, D21 and D28 (marked with a red star). For experiment 2, hatched chicks received either a commercial starter diet (control) or a commercial diet enriched with a β-glucan additive (β-glucan). Chicks received at 28 days of age (DPI D0), the immunization with HuSA. Blood was collected at DPI: D0, D7, D14, D21 and D28 (marked with a red star). Figure created with BioRender.com.

Transgenerational effects of maternal immune activation were studied by immunizing the hens with either PBS, HuSA or LPS. For experiment 1, the effects on the offspring specific antibody response were evaluated by immunization with HuSA or HuSA+LPS followed by measuring HuSA-specific IgM and IgY antibody titers in blood. A schematic design of the experiment is shown in Figure 1. In experiment 1 hens were randomly divided into three maternal groups (PBS, HuSA or LPS) with 15 individually housed hens per maternal group. During egg collection a total of 150 eggs of each maternal group were collected, incubated and hatched. A total of 60 female chicks, with 20 chicks per maternal group, were randomly selected and randomly divided over 2 pens. Each pen represents one of the two immunization groups (HuSA or HuSA+LPS). For experiment 2, the effects on the offspring specific antibody response were evaluated by immunization with HuSA followed by measuring HuSA-specific IgM and IgY antibody titers in blood (Figure 1). Experiment 2 also includes a dietary treatment, which started directly post hatch until the end of the experiment. A schematic design of the experiment is shown in Figure 1. Until hatch, design of experiment 2 was similar to that of experiment 1. Hereafter, a total of 60 female chicks, with 20 chicks per maternal group, were divided into 3 diet challenge groups, with 5 replicates per group. Entire offspring received an immunization with HuSA.

### Chickens, Housing, Management and Treatments

Two independent experiments were performed using commercial purebred White Leghorn chickens (WA chicken line; Hendrix Genetics B.V., Boxmeer, the Netherlands). For both experiments the chickens were kept according to standard management guidelines of Hendrix Genetics, which was previously described by Van der Klein et al. (18).

#### Experiment 1

Thirty-week-old hens were individually housed in wired cages of 0.6 m^2^. The hens were randomly divided in 3 groups of each 10 hens. After 3 weeks habituation the hens were intratracheally (i.t.) immunized with: 0.5 ml PBS (control; Group PBS), 1 mg HuSA (Sigma-Aldrich corporations, St. Louis, MO, USA) in 0.5 ml PBS (Group HuSA) or 1 mg LPS (*Escherichia coli* serotype O55:B5, L2880, Sigma-Aldrich) in 0.5 ml PBS (group LPS). The i.t immunizations were performed by placing a 1.2 × 60 mm blunted anal cannula (InstruVet, Cuijk, the Netherlands), on a 1-mL syringe, gently into the trachea of the chick (19). At the day of immunization all hens were inseminated with pooled sperm and this insemination was repeated after 2 weeks. In the third week after priming, which is 1 week after the second insemination, collection of fertilized eggs was started and 10 eggs per hen were collected during a period of 2 weeks. All eggs were marked per individual hen and stored at room temperature until incubation. Eggs were incubated at research facility “Carus” of Wageningen University & Research according to standard production practices. The newly hatched chicks were sexed visually by wing feather sexing and 20 hens per maternal immune activation group were randomly selected and equally divided over 2 pens. Both pens contained chicks of all maternal treatments. All chicks within a treatment group were from different mother hens. These treatment groups were at 4 weeks post hatch i.t. immunized, as described earlier, with either: 1 mg HuSA in 0.5 ml PBS (group HuSA) or 1 mg HuSA + 1 mg LPS in 0.5 ml PBS (group HuSA+LPS). At day 0, 1, 7, 14, 21 and 28 post immunization 1 ml heparinized blood was collected from the brachial wing vein. Blood was centrifuged (5,250 x g, 10 min at room temperature). Plasma was collected and stored at −20°C until use.

#### Experiment 2

Thirty-week-old hens were individually housed in wired cages of 0.6 m^2^. These hens were divided in 3 groups of each 15 hens. After 3 weeks habituation the hens were intratracheally (i.t.) immunized, as described earlier, with: 0.5 ml PBS (control; Group PBS), 1 mg LPS (Sigma Aldrich) in 0.5 ml PBS (group LPS) or 1 mg HuSA (Sigma Aldrich) in 0.5 ml PBS (Group HuSA). At the day of immunization all hens were inseminated with pooled sperm and this insemination was repeated after 2 weeks. The collection of fertilized eggs was started 1 week after the second insemination and per hen 10 eggs were collected during a period of 2 weeks. All eggs were marked per individual hen and stored at room temperature until incubation. Newly hatched chicks were sexed and 20 hens per maternal immune activation group were selected. The in total 60 hens were allocated to 10 pens. These pens were randomly divided into 2 groups of 5 pens. Each group with 5 pens received either a commercial starter diet (control) or a commercial starter diet enriched with a β-glucan additive (250 ppm, Macrogard, Orffa, Werkendam, the Netherlands). All hens were at 4 weeks post hatch i.t. immunized, as described earlier, with 1 mg HuSA in 0.5 ml PBS. At day 0, 7, 14, 21 and 28 post immunization 1 ml heparinized blood was collected from the brachial wing vein. Blood was centrifuged (5,250 x g, 10 min at room temperature) and plasma was collected and stored at−20°C until use.

### Detection of IgM and IgY Antibodies Binding HuSA

Titers of HuSA-specific IgM and IgY antibodies were determined in individual plasma samples by an indirect two-step ELISA as described previously (20, 21). Briefly, flat bottomed 96-wells plates (Greiner Bio-One, Alphen aan den Rijn, the Netherlands) were coated with 100 μL of 0.1 M carbonate buffer (pH 9.6) containing 4 μg/mL HuSA (Sigma-Aldrich), incubated overnight at RT followed by a washing step. All washing steps during this ELISA assay were done with tap water containing 0.05% Tween ® 20 (Sigma-Aldrich). The washed plates were incubated for 90 min at RT with 4-step serial dilutions of serum samples in PBS containing 0.05% Tween ® 20, and 0.5% normal horse serum in duplicate. After washing, plates were incubated again for 90 min at RT with 100 μL of a 1:40.000 dilution of goat-anti-chicken IgY_Fc_ (Bethyl Laboratories Inc, Texas, USA) or a 100 μL 1:20.000 dilution of goat-anti-chicken IgM (Bethyl Laboratories Inc) in PBS containing 0.05% Tween ® 20 and 0.5% normal horse serum. After washing, plates were incubated with tetramethylbenzidine and 0.05% H_2_O_2_ at RT and after 10 min the reaction was stopped with 1.25% H_2_SO_4_. The optical density was measured at 450 nm with a spectrophotometer (Multiscan™, Thermo Fisher Scientific, Waltham, MA, USA). Titers represents levels of antibodies relative to a standard positive control blood plasma sample. Titers were calculated as described by Berghof et al. (22).

### Statistical Analysis

Statistical analysis was done in SAS v 9.4 (SAS software by SAS institute INC.). All statistical analysis were performed with the PROC MIXED procedure. For both experiments the statistical model used for estimating maternal immune activation differences on antibody titers based on repeated observations in the offspring was as follows:


$$\begin{array}{l} Y_{ijkl}=\mu \;+\;Treatment\;1_{i}+Treatment\;2_{j}+Day_{k}+\;{\left(T1\;x\;D\right)}_{ik}\\ +\;Chicken_{l}\;+\;e_{ijkl} \end{array}$$

where *Y*_*ijkl*_is the HuSA binding IgM or IgY titer of offspring chickens, Treatment 1_i_ is the fixed effect for maternal immune activation (i = HuSA, LPS or PBS), Treatment 2_j_ is the fixed effect for offspring treatments (j = HuSA, HuSA + LPS in experiment 1 and control diet or ß-glucan diet in experiment 2), Day_k_ is the fixed effect of day (post immunization) at which the blood was collected for antibody measurement (k = 0, 1, 7, 14, 21, 28), *(T1 x D)*_*ik*_ is the fixed effect of the interaction between Treatment 1_i_ (T1) and Day_k_ (D), Chicken_l_ is the random effect of the l_th_ chicken (l = 1–54) and *e*_*ijkl*_ is the residual term which was tested for approaching normality. The compound symmetry structure was used as covariance structure. *Post-hoc* pair wise comparisons were corrected by Tukey-Kramer adjustment.

## Results

### Experiment 1: Effect of Maternal Immune Activation on Antibody Production in Offspring

**Figure 2:**
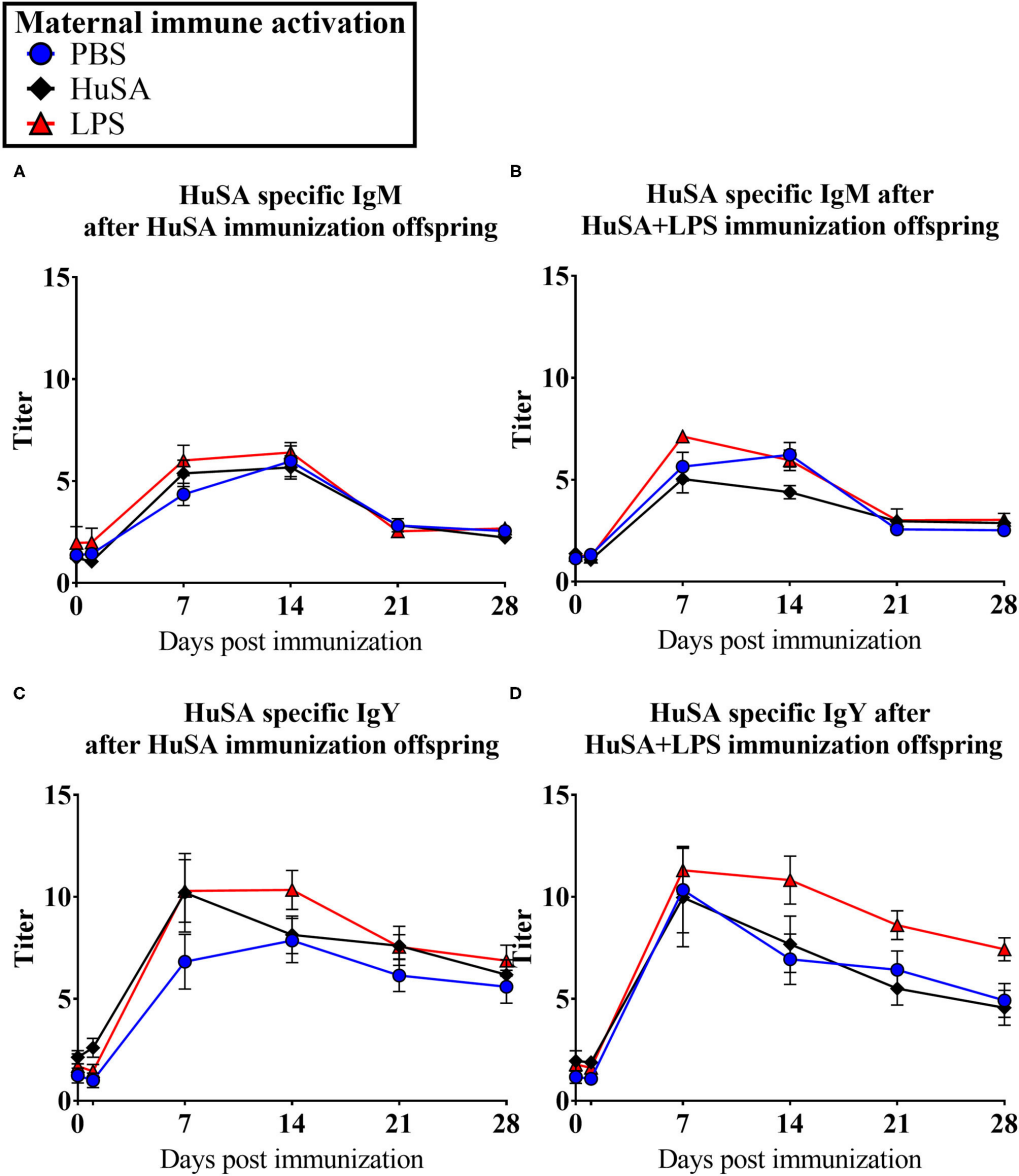
Results of experiment 1 with IgM and IgY anti-HuSA titers of chicks originating from immune activated hens with either PBS, HuSA or LPS. The chicks were immunized 4 weeks post hatch with either HuSA or HuSA+LPS. Titers were measured at 0, 1, 7, 14, 21 and 28 days post immunization. **(A)** anti-HuSA IgM titers after HuSA immunization. **(B)** anti-HuSA IgM titers after HuSA+LPS immunization **(C)** anti-HuSA IgY titers after HuSA immunization. **(D)** anti-HuSA IgY titers after HuSA+LPS immunization. Data are presented as means ± SEM, and *N* = 20 chickens per group.

Effects of maternal immune activation (PBS, HuSA, or LPS, respectively) on HuSA specific antibodies were measured in the offspring after immunization with either HuSA or HuSA+LPS at 4 weeks of age. Titers were measured at day 0, 1, 7, 14, 21 and 28 post immunization and the least-squares means (LSmeans) of the titers are presented in Table 1. Overall, the average antibody titers of both isotypes increased from day 0 to day 7 followed by a gradual decrease after day 7 onwards, indicating a specific antibody response to HuSA (*p* < 0.001; Table 1). Comparison of the HuSA and HuSA+LPS responses in the offspring revealed no differences on HuSA titers and showed similar kinetics in time (Figures 2A–D). Therefore, HuSA and HuSA+LPS treatments were combined in the further statistical analysis. Maternal immune activation with LPS enhanced IgM responses to HuSA in the offspring, compared to offspring of the HuSA immunized mother hens (titers 3.6 and 3.0; *P* < 0.05) (Table 1). Maternal immune activation with LPS resulted in higher IgY anti-HuSA titers in the offspring compared to offspring of the PBS treated mother hens (titers 6.9 and 5.1; *P* ≤ 0.05; Table 1).

**Table 1:** Effect of maternal immune activation on HuSA specific IgM and IgY titers during 4 weeks after i.t. immunization of offspring at 4 weeks of age.

	**Isotype**	**Maternal treatment** ^**1**^				**Main effects (** ***P*** **-value)**		
		**PBS**	**HuSA**	**LPS**	**SEM**	**Time**	**Treatment**	Time ^*^Treatment^**4**^
Anti-HuSA	IgM^2^,^3^	3.2^ab^	3.0^b^	3.6^a^	0.2	<0.001	0.039	0.049
	IgY^2^,^3^	5.1^b^	5.6^ab^	6.9^a^	0.7	<0.001	0.030	0.024

1 *Maternal treatment: i.t. immune activation of hens: 1 mg HuSA or 1 mg LPS in 0.5 ml PBS*.

2 *Least squares means of i.t. immunization HuSA and HuSA+LPS combined*.

3 *Least square means within a row lacking a common superscript (a, b or ab) are significantly different (P < 0.05)*.

4 *Interacting variables are represented with an asterisk*

(*).

### Experiment 2: Effect of Maternal Immune Activation on Antibody Production and Dietary β-Glucan Modulation in Offspring

**Figure 3:**
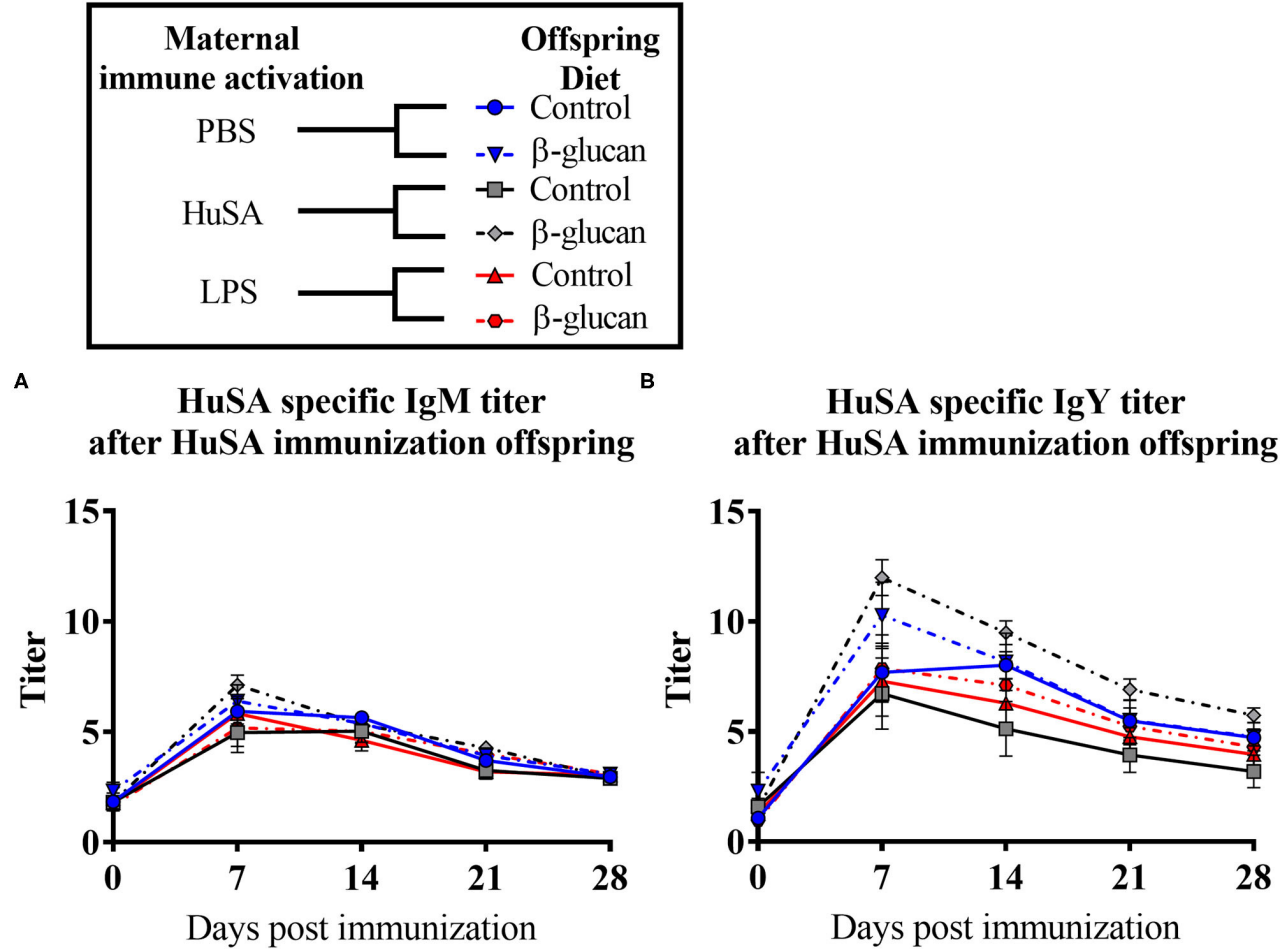
Results of experiment 2 with IgM and IgY anti-HuSA titers of chicks originating from hens immunized with either PBS, HuSA or LPS. The chicks were fed either the control diet or the diet enriched with β-glucan. Chicks were immunized 4 weeks post hatch with HuSA and titers were measured at 0, 3, 7, 14, 21 and 28 days post immunization. **(A)** anti-HuSA IgM titers after HuSA immunization. **(B)** anti-HuSA IgY titers after HuSA immunization. Data are presented as means ± SEM, and *N* = 10 chickens per group.

Effects of maternal immune activation with PBS, HuSA or LPS on IgM and IgY anti-HuSA antibody titers were measured in the offspring after HuSA immunization with and without administration of dietary β-glucan. Titers of HuSA specific IgM and IgY antibodies were measured at day 0, 3, 7, 14, 21 and 28 and the LSmeans of the titers during this time period are shown in Table 2. Antibody titers of both isotypes increased in time, indicating a specific antibody response to HuSA (*p* < 0.001; Figure 3). Both graphs in Figure 2 show similar kinetics with highest titers measured 7 days post immunization whereafter the titers decline. In contrast to experiment 1, no differences in IgM and IgY anti-HuSA antibodies were observed upon maternal immune activation with PBS, HuSA, or LPS in offspring without dietary treatment (Table 2).

**Table 2:** Effect of dietary β-glucan on HuSA specific IgM and IgY titers in offspring after primary i.t. immunization with HuSA at 4 weeks of age.

	**Isotype**	**PBS**		**HuSA**		**LPS**		**SEM**	**Main effects (*****P-*****value)**^**1**^, ^**2**^					
		**Control**	**β-Glucan**	**Control**	**β-Glucan**	**Control**	**β-Glucan**		**Time**	**Treatment 1**	**Treatment 2**	Treatment 1 ^*^Treatment 2	Time ^*^Treatment 1	Time ^*^treatment 2^**5**^
Anti-HuSA	IgM^3^,^4^	4.00	4.20	3.60	4.30	3.70	3.60	0.20	<0.001	0.228	0.206	0.351	0.870	0.345
	IgY^3^,^4^	5.40^ab^	6.21^ab^	4.12^**b**^	7.13^**a**^	4.72^ab^	4.80^ab^	0.60	<0.001	0.193	0.011	0.049	0.500	0.005

1 *Treatment 1: i.t. immune activation of mother hens: 1 mg HuSA and 1 mg LPS in 0.5 ml PBS*.

2 *Treatment 2: Dietary intervention with or without β-glucan enrichment in offspring; n = 5 pens per group*.

3 *Least squares means*.

4 *Least square means within a row lacking a common superscript (a, b or ab) are significantly different (P < 0.05)*.

5 *Interacting variables are represented with an asterisk*

(*).

Next, the effect of a dietary treatment with β-glucan was investigated. No main interaction effect between maternal immune activation and dietary treatment or main effects for maternal or dietary treatments on IgM were found. However, for IgY, an interaction was found between maternal immune activation and dietary treatment (*P* < 0.05; Table 2). Maternal immune activation with HuSA followed by a β-glucan dietary treatment in the offspring resulted in a higher average IgY anti-HuSA antibody titer compared to chicks that obtained the standard diet (titers 7.13 and 4.12; *P* < 0.05; Figure 3 and Table 2). Maternal immune activation with PBS followed by a β-glucan dietary treatment in the offspring resulted numerically in a higher average IgY anti-HuSA antibody titer compared to chicks that obtained the standard diet (titers 6.21 and 5.40; n.s.; Table 2). In contrast, maternal immune activation with LPS followed by a β-glucan dietary treatment showed no difference compared to the same chicks that obtained the standard diet (titers 4.80 and 4.72; n.s.; Table 2).

**Figure 4:**
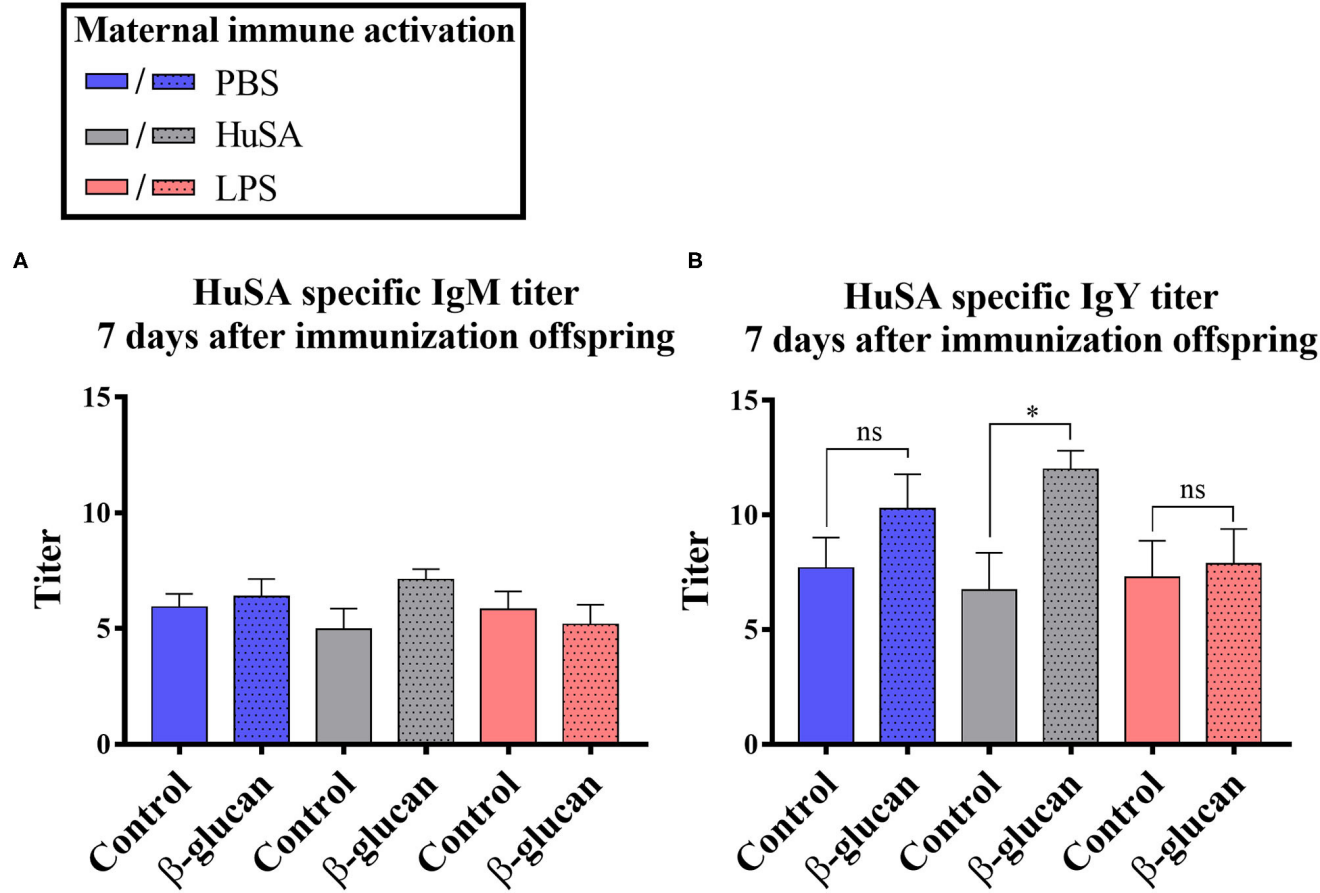
Results of experiment 2 with IgM and IgY anti-HuSA titers of chicks originating from hens immunized with either PBS, HuSA or LPS. The chicks were fed either the control diet or the diet enriched with β-glucan. Data of offspring 7 days post immunization are presented. **(A)** IgM anti-HuSA titers 7 days after the HuSA immunization and **(B)** IgY anti-HuSA titers 7 days after the HuSA immunization. Each bar represents means ± SEM, and *N* = 10 chickens per group. Effects are represented as ^*^ and were considered to be significant when *P* ≤ 0.05. ns, not significant.

Furthermore, an interaction was found between time and β-glucan dietary intervention for IgY anti-HuSA antibody titers (*P* < 0.05; Table 2). Figures 4A,B show the IgM- and IgY-specific HuSA titers at day 7, the time point where the antibody levels reached their maximum. In accordance with the results shown in Table 2, the HuSA titers at time point day 7 show that maternal immune activation with HuSA followed by a β-glucan dietary treatment in the offspring resulted in a higher average IgY anti-HuSA antibody titer compared to the control group that obtained the standard diet (titers 11.99 and 6.73; *P* < 0.05; Figure 4B). Offspring originating from the LPS treated mother hens showed no difference with the control group that obtained the standard diet (titers 7.86 and 7.29; n.s.; Figure 4B). Maternal immune activation with PBS followed by a β-glucan dietary treatment in the offspring resulted numerically in a higher average IgY anti-HuSA antibody titer compared to the same chicks that obtained the standard diet (titers 10.28 and 7.69; n.s.; Figure 4B). When comparing the three control offspring groups that obtained the standard diet, no differences in anti-HuSA titers were found (titers 7.69, 6.73 and 7.29; n.s.; Figure 4B).

## Discussion

In this study, we investigated whether maternal innate and adaptive immune activation affected the specific antibody responses in the next generation. Two experiments were conducted to investigate whether specific and innate immunogenic stimulation of the hen, i.e., HuSA and LPS, respectively, alter the offspring's specific antibody response. Furthermore, maternal innate immune activation by LPS seems to influence immune modulation by dietary β-glucans in the next generation. Although the results were not fully consistent, maternal immune activation with LPS appeared to have clear effects on the specific antibody responses in both experiments.

Within the current study, intratracheal immunization was used to mimic the practical circumstances of poultry in barns. Chickens inhale substantial amounts of endotoxins and bacterial numbers present in dust particles in poultry barns, including the *E. coli* (23–25). *E. coli* causes frequently infections in poultry and infect chickens mainly via the respiratory tract and intestines. LPS is a major component of the outer membrane of Gram-negative bacteria, such as *E. coli*. In the current study we used LPS, from the *E. coli* serotype O55:B5, intratracheally and is therefore a natural route to exposure of antigens, a good model to investigate effects on animal health and translatable to the practical circumstances. Intratracheal immunization with LPS and HuSA was previously successfully performed to initiate a substantial systemic immune response. Serological parameters, such as antibody titers, were affected and proven to be a good read out parameter (19, 24, 26). Another study with chickens shows that intratracheally applied beads were found back in lungs, bursa, air sacs, humerus and radius (27), indicating that intratracheally administered antigens are easily absorbed.

In experiment 1, maternal immune activation with LPS leads to an increase in HuSA-specific IgY responses of the offspring. This observation with LPS suggests a transgenerational relation between the innate immune system of the hen and the immune system of the offspring. Our results are in accordance with a study in pied flycatchers, where maternal immune activation with LPS was found to have transgenerational effects resulting in elevated antibody production in the offspring (9).

The results from experiment 1 were not completely confirmed by those from experiment 2. Maternal i.t. immune activation with either specific antigen HuSA or innate antigen LPS did not increase the antigen specific IgY response against HuSA in the offspring. However, for IgY, an interaction was found between maternal immune activation and dietary treatment (*P* < 0.05; Table 2). This implies that maternal immune activation influenced the stimulating effect of dietary β-glucans on the specific antibody response in the offspring. Dietary β-glucans are known to enhance antibody responses in chicken, (13, 28, 29). In the current study however, we show that chicks originating from the LPS immune activated hens were unresponsive to the dietary β-glucans. Thus, maternal immune experiences, such as infections or vaccinations, may influence the effects of dietary interventions with feed additives on the immune system in the offspring. This observation might indicate that maternal immune activation with LPS leads to tolerance of innate immune cells to β-glucans in the offspring, resulting in reduced HuSA specific IgY production. To prove this, future studies should therefore focus on the effects of maternal immune activation on antigen presentation and B cell activating coreceptors on antigen presenting cells.

Both independent experiments with laying hens suggest that, despite the variation in outcomes, immunological experiences of the mother influence specific antibody production in the offspring. Both experiments demonstrated a memory-like mechanism, influenced by the maternal immune system stimulated with LPS. The effect on specific IgY antibody responses in the offspring caused by innate immune stimulation suggests interaction between maternal innate and neonatal immune system. These results could indicate the presence of transgenerational trained innate immunity where innate stimulation has induced a poly-specific memory like function which is known as one of the main consequences of trained innate immunity.

In recent years much attention has been paid to this concept of trained innate immunity. Studies in a broad variety of animal species, including mammals, birds and fish, have shown that the innate immune system can be trained, indicating the conserved nature of this immune mechanism (30–32). Trained innate immunity is defined as the activation of the innate immune system resulting in a memory-like enhanced responsiveness to subsequent triggers driven by epigenetic mechanisms and metabolic reprogramming. The training by innate antigens leads to an increased resistance to infections (33, 34). This effect is long-lasting and is polyspecific, i.e., one innate antigenic stimulus can lead to an amplified response against other unrelated stimuli (35). It was also described that there is a dynamic interaction between LPS and β-glucan by inducing and damping tolerance (36). This was caused by DNA epigenetic changes of the H3K27ac and H3K4me markers and part of the trained innate immunity concept. LPS is an important activator of innate immune cells, such as monocytes, macrophages and dendritic cells in a wide variety of animal species, and plays an important role within trained innate immunity and related epigenetic effects (30, 37). The transgenerational effects found in experiment 1 and experiment 2 could be explained by epigenetic effects of the innate immune system, which was already previously proposed by Berghof et al. (5). Studies have shown that trained innate immunity is maintained by epigenetic mechanisms such as DNA modification (36). These modifications influence the activity of immune-related genes in innate immune cells (37). For example, *in vitro* trained chicken macrophages showed enhanced expression of B cell activating molecule CD40 (38). Thus, trained macrophages may enhance B cell activation *in vivo* as well, resulting in enhanced antibody responses.

Transfer of epigenetic DNA modifications to next generations has indeed been observed in chickens (39). The transgenerational epigenetic effects can be found in the F_1_ and even F_2_ generation (40). It is therefore reasonably to suggest that components that influence the innate immune system of the mother animal, like LPS did in the current study, influence the maturation of the neonatal immune system in a transgenerational manner and as a consequence the disease resistance of the neonate (41). The concept of trained innate immunity could fit well in the current study and is therefore proposed as an explanatory concept. However, epigenetic data supporting this theory is lacking in the current study. More research on this is needed investigate this new concept.

Especially the results of experiment 2 suggests that maternal immune activation with LPS leads to tolerance of innate immune cells to β-glucans in the offspring, resulting in a lack of HuSA specific IgY antibody production. On the other hand, anti-HuSA maternal antibodies, which may influence the embryonic development or may be present in the HuSA-group offspring of experiment 2, did not result in tolerance. This could have been expected, because tolerance formation against antigens is blocked when antigen specific maternal antibodies are present as described previously (42, 43). Presence of substantial amounts of HuSA specific maternal antibodies is however unlikely. Because fertilized eggs were collected 3–5 weeks after HuSA immune activation. The level of HuSA specific maternal antibodies in the offspring is then likely negligible. Indeed, no differences of antibody titers at day 0 were observed for the different maternal treatment groups. However, we cannot completely rule out that HuSA specific maternal antibodies induced an imprinting effect before day 0 on development of the neonate. This should be addressed in future studies and more research on this is needed. The main goal of this study was whether there are transgenerational effects of maternal immunization. Remains of LPS in the hen's body was therefore not measured in hens. However, it is very likely that LPS has been enzymatic degraded and removed out of the chicken's body before fertilization and egg collection took place. In mice it has been found that LPS has a half-life of 3–4 min in the blood and that enhanced innate pro-inflammatory responses were back to normal within 4 h (44, 45). Also a study with chickens show that the enhancing effect of LPS administration in the trachea on TLR4 expression were back to original state within 72 h (46). Fertilized eggs were collected 3–5 weeks after maternal immune activation, it is therefore highly plausible to expect that LPS was not present in the yolk.

The current study did not elucidate how the transgenerational transmission of this immunity takes place. A highly plausible possibility is that the activity of immune related genes in germline DNA of the embryo is influenced by DNA methylation, histone acetylation or formation of regulatory micro-RNA molecules (39, 40). Especially this transfer of epigenetic modifications deserves the consideration for investigation to unravel the mechanisms that causes effects found in the present study. More read-out parameters which have already described to be associated in visualizing trained innate immunity and transgenerational epigenetics in mammals, including histone modification analyses or transcriptomics, may be worth considering (41, 47). The inconsistency between the outcomes of both experiments could be explained by the biological variation and/or life history of the hens whereby environmental confounding factors such as temperature, hygienic circumstances and the effects of intestinal microbiota should not be ignored (48, 49). This indicates the importance of unraveling the mechanisms that are influencing transgenerational inheritance. More knowledge about transgenerational effects of maternal immune activation or infection will contribute to a better understanding of the variation in immune phenotypes, disease resistance and metabolic disorders. Knowledge that may have consequences for the animal husbandry sector how to optimize their vaccination strategies and how to apply immunomodulatory dietary additives more effectively.

## Data Availability Statement

The raw data supporting the conclusions of this article will be made available by the authors, without undue reservation.

## Ethics Statement

The animal study was reviewed and approved by Animal Welfare Committee of Wageningen University and Research.

## Author Contributions

MV, HP, and AL organized the database. AL and HP performed the statistical analysis. MV and AL wrote the first draft of the manuscript and contributed to the visualization of the data. HP and AL contributed to the funding acquisition of the project. JA, HP, and AL contributed to the field work and primary measurements. All authors contributed to conception, design of the study, manuscript revision, read, and approved the submitted version.

## Funding

This study was financially supported by the Dutch Animal Feed Research Consortium (Vereniging Diervoederonderzoek Nederland).

## Conflict of Interest

The authors declare that the research was conducted in the absence of any commercial or financial relationships that could be construed as a potential conflict of interest.

## Publisher's Note

All claims expressed in this article are solely those of the authors and do not necessarily represent those of their affiliated organizations, or those of the publisher, the editors and the reviewers. Any product that may be evaluated in this article, or claim that may be made by its manufacturer, is not guaranteed or endorsed by the publisher.
